# Supplementary material for: Simultaneous Detection of RIG-1, MDA5, and IFIT-1 Expression Is a Convenient Tool for Evaluation of the Interferon-Mediated Response
Source: Viruses. 2022 Sep 21;14(10):2090. doi: 10.3390/v14102090 (PMC9609481; doi:10.3390/v14102090)
Supplement: Supplementary file 1 [file viruses-14-02090-s001.zip › viruses-1866308-SI.pdf]

Simultaneous Detection of RIG-1, MDA5, and IFIT-1 Expression Is a Convenient Tool for Evaluation of the Interferon-Mediated Response

Supplementary files

Table S1. Primers and probes

Target	Position	Sequence 5’-3’
**RIG-1 (DDX58)** *NM_014314.4** *NM_001385907.1* *NM_001385910.1* *NM_001385912.1* *NM_001385913.1* *NM_001385914.1*	qH_RIG1_F1940	GAGCACTTGTGGACGCTTTA
	qH_RIG1_R2053	ATACACTTCTGTGCCGGGAG
	qH_RIG1_O2002	(ROX)-CCTGGCATATTGACTGGACGTGGC-(BHQ2)
**MDA5 (IFIH-1)** *NM_022168.4*	qH_IFIH-1_F2667	AAACCCATGACACAGAATGAACA
	qH_IFIH-1_R2864	TGTGAGCAACCAGGACGTAG
	qH_IFIH-1_O2744	(Cy5.5)-CACAGTGGCAGAAGAAGGTCTGGA-(BHQ3)
**IFIT-1** *NM_001548.5** *NM_001270927.2* *NM_001270928.2* *NM_001270929.2* *NM_001270930.2*	qH_IFIT-1_F1376	AAACTTCGGAGAAAGGCATTAGAT
	qH_IFIT-1_R1540	TGAAATGAAATGTGAAAGTGGCTG
	qH_IFIT-1_O1479	(HEX)-CCTGAGACTGGCTGCTGACTTTGAGAAC-(BHQ1)
**ANXA-1** *NM_000700.3*	qH_ANXA-1_F750	ACCACCAGAAGCTATCCACAA
	qH_ANXA-1_R928	CGAGTTCCAACACCTTTCATGG
	qH_ANXA-1_O879	(Cy5.5)-AAGTGCGCCACAAGCAAACCAGC-(BHQ3)
**poly IFNA** *NM_024013.3** *NM_000605.4* *NM_021068.4* *NM_002169.3* *NM_021002.2* *NM_021057.2* *NM_002170.4* *NM_002171.2* *NM_006900.4* *NM_002172.3* *NM_002173.3* *NM_021268.2* *NM_002175.2*	qH_IFNA_F294	AGCCATCTCTGTCCTCCATGAG
	qH_IFNA_R580	TTTCTGCTCTGACAACCTCCC
**IFNB** *NM_002176.4*	qH_IFNB1_F227	TGCCTCAAGGACAGGATGAACT
	qH-IFNB1_R405	TTAGCCAGGAGGTTCTCAACAATAG
	qH_IFNB1_O285	(HEX)-TCCAGAAGGAGGACGCCGCATTGAC-(BHQ1)
**IFNL2/3**	qH_IFNL2/3_F347	GAGGGCCAAAGATGCCTTAGAA

NM_172138.2*	qH_IFNL2/3_R518	AGTGTCAGCGGTGGCCT
NM_001346937.2<br>***NM_172139.4***	qH_IFNL2/3_O453	(HEX)-GCAGCTGCAGGTGAGGGAGC-(BHQ1)
**MxA**<br>***NM_001144925.2****	qH_MxA_F2345	GAGACAATCGTGAAACAGCAAATCA
	qH_MxA_R2449	TATCGAAACTCTGTGAAAGCAAGC
NM_002462.5<br>***NM_001178046.3***	qH_MxA_O2373	(HEX)- CACTGGAAGAGCCGGCTGGGATATG-(BHQ1)
**SOCS-1**<br>***NM_003745.2***	qH_SOCS-1_F1030	CCTGGTTGTTGTAGCAGCTTA
	qH_SOCS-1_R1132	CCTGGTTTGTGCAAAGATACTG
	qH_SOCS-1_O1093	(ROX)-CCTGGTTGTTGTAGCAGCTTA-(BHQ2)
**ACTB**<br>***NM_001101.5***	qH_ACTB_F353	TCTACAATGAGCTGCGTGTGGCTCCC
	qH_ACTB_R489	AGCAACGTACATGGCTGGGGTGTTGAA
	qH_ACTB_O419	(FAM)-<br>CCAAGGCCAACCGCGAGAAGATGACCCAGAT-CAT-<br>(BHQ1)
**GAPDH**<br>***NM_002046.7****	qH_GAPDH_F23	CAGTCAGCCGCATCTTCTTTTGCGTCG
	qH_GAPDH_R152	CAGAGTTAAAAGCAGCCCTGGTGACCAG
NM_001289745.3<br>***NM_001289746.2***<br>***NM_001357943.2***	qH_GAPDH_78	(FAM)-<br>TGGGGAAGGTGAAGGTCGGAGTCAACGGATTTG-<br>GTC-(BHQ1)

* primers and probe positions are specified in the sequence of this transcript

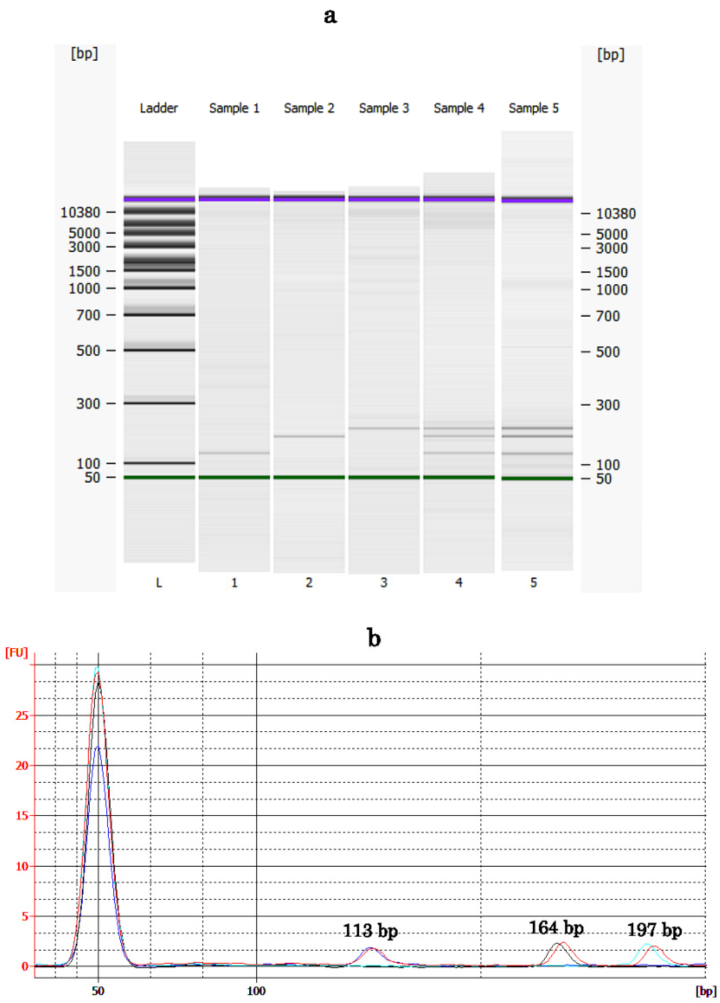

Figure S1. Three specific amplicon products (RIG-1, IFIT, MDA5) are amplified by the developed multiplex qPCR assay. (a) Capillary electrophoresis. Samples: 1 – RIG-1 monoplex (113 bp); 2 – IFIT monoplex (164 bp); 3 – MDA5 monoplex (197 bp); 4 – mix of monoplex products; 5 – multiplex reaction products. (b) electrophoregram showing product peaks and their proper coincidence: RIG-1 (dark blue); IFIT (green); MDA5 (light blue); and multiplex PCR (red).

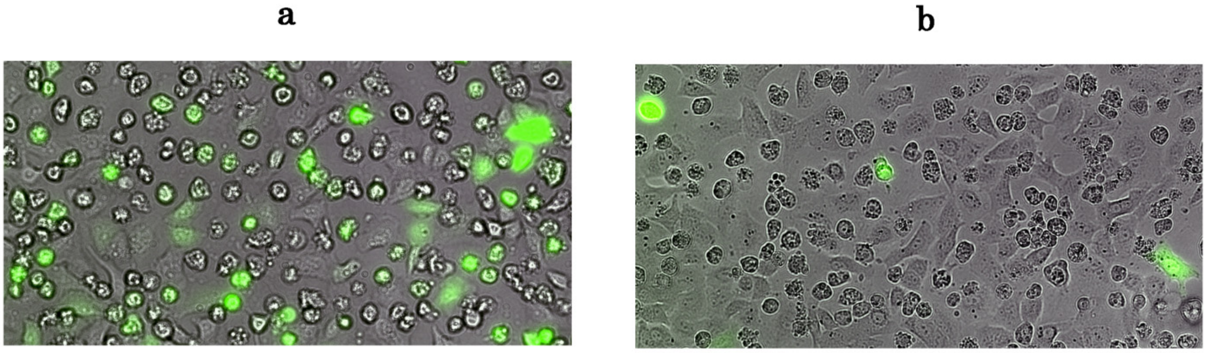

Figure S2. Transfection of A549 cells with synthetic RNA coding GFP leads to stable translation 24 hours after transfection. (a) live cell microscopy of A549 cells transfected with IVT-RNA-GFP. (b) live cell microscopy of A549 cells transfected with IVT-saRNA-GFP

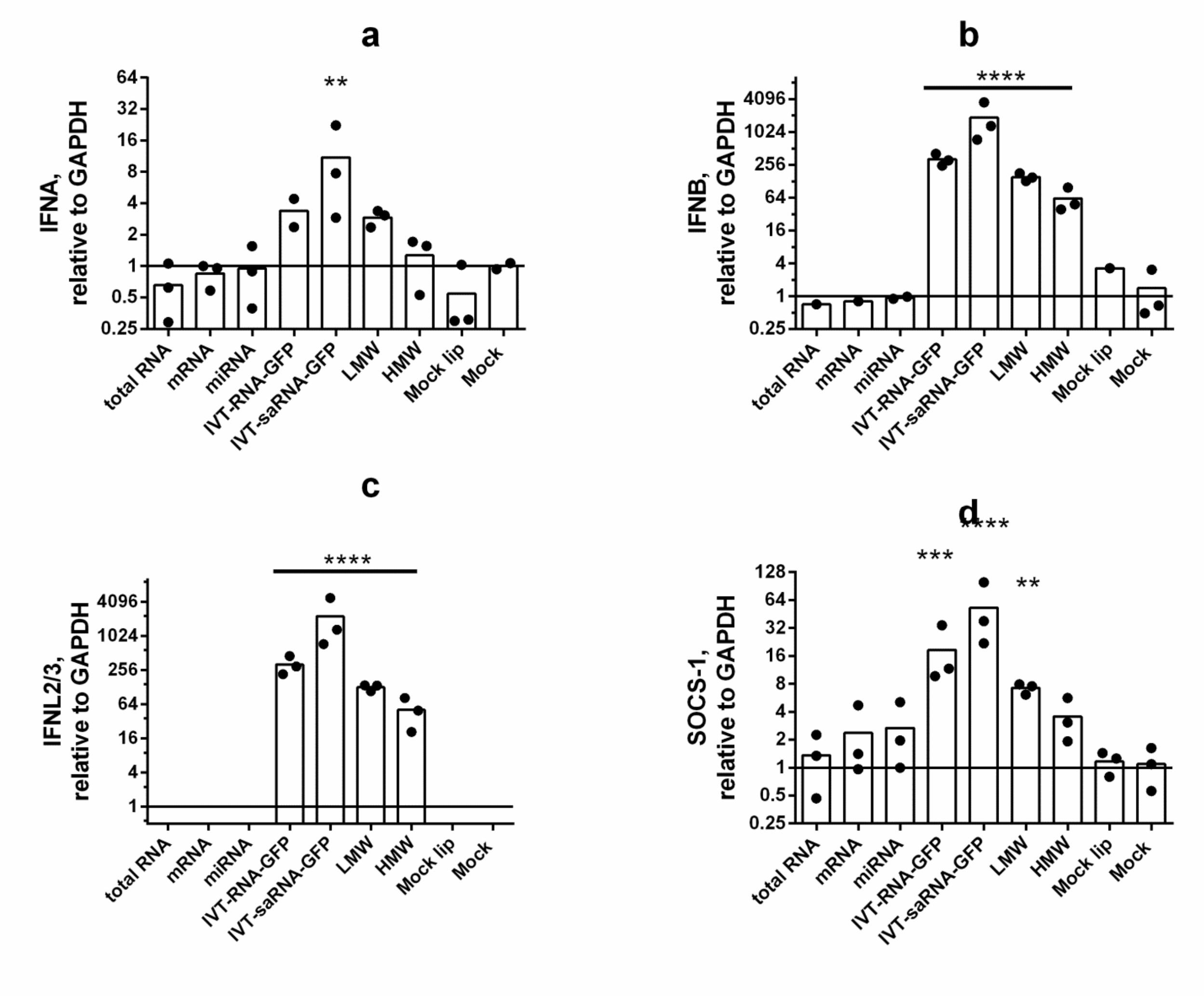

Figure S3. Synthetic RNAs induce IFN expression. Expression levels of IFNA **(a)**, IFNB **(b)**, IFNL2/3 **(c)**, and SOCS-1 **(d)** were analyzed at 24 hours post transfection. Gene expression was analyzed via the ΔΔCt method (relative to GAPDH). Statistical significance (p-value) was determined by ordinary one-way ANOVA, followed by a pairwise Holm-Sidak's multiple comparisons test: **** — adjusted P Value < 0.0001; *** — < 0.001; ** — < 0.01 compared to Mock. Mock – intact cells that were cultured in the same conditions and were not transfected (instead, sterile medium F12K was added). At least three biological replicates were used for each experimental data point. Data are represented as median.

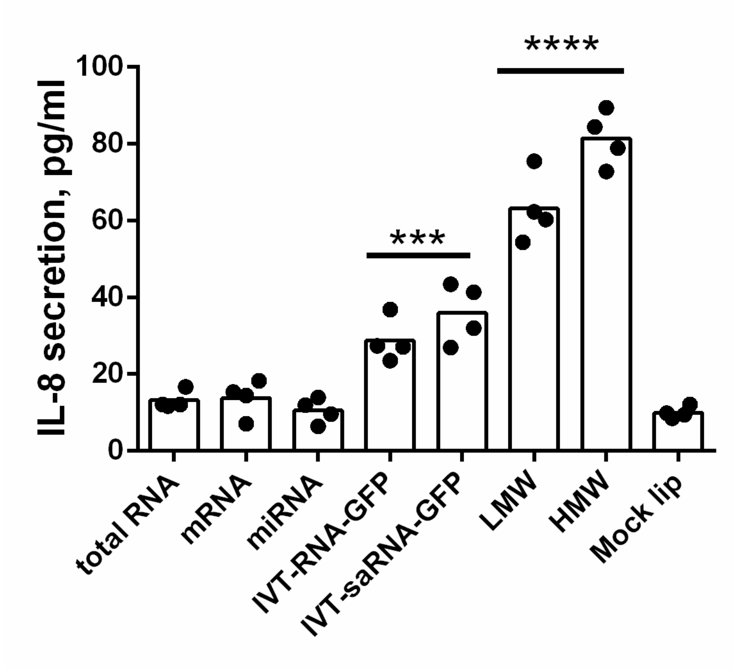

Figure S4. IL-8 secretion is elevated in response to exogenous RNA. IL-8 levels in supernatants were measured by ELISA 24 hours post transfection. Statistical significance (p-value) was determined by ordinary one-way ANOVA, followed by Holm-Sidak's multiple comparisons test: **** — adjusted P Value < 0.0001; *** — < 0.001 compared to Mock lip. Mock lip – cells that were cultured in the same conditions and were transfected only with lipofectamin and sterile medium. At least three biological replicates were used for each experimental data point. Data are represented as mean ± SD.

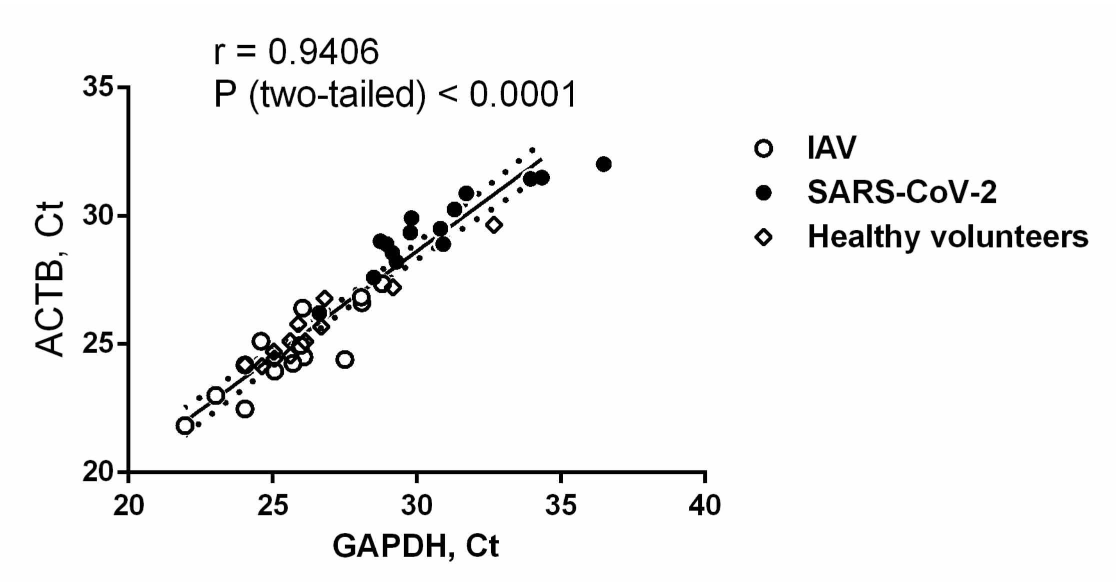

Figure S5. The selected reference genes, GAPDH and ACTB, are in good correlation to each other. GAPDH Ct values are indicated on the x-axis; ACTB Ct values are indicated on the y-axis (a). Spearman correlation coefficient and P value are shown on the graph.

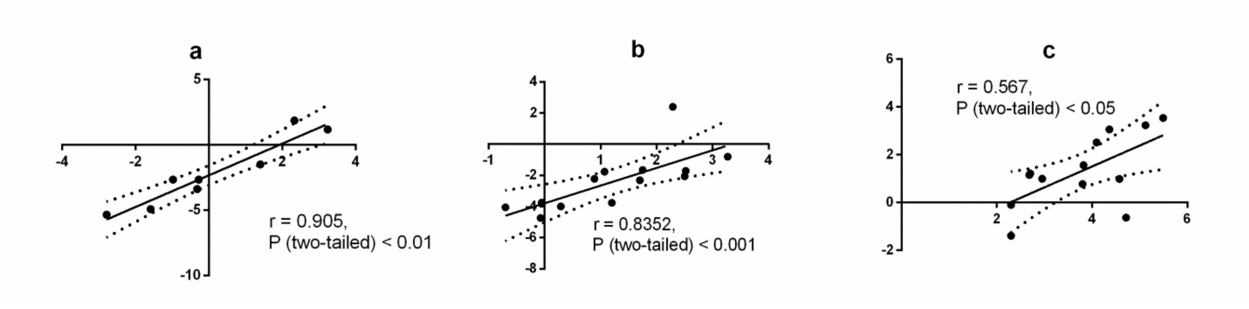

Figure S6. Correlation between IFIT-1 and RIG-1 expression with COVID-19 (a), influenza (b), or healthy volunteers (c) at the early phase of illness (3-4 days post symptoms onset). RIG-1 delta Ct values are indicated on the x-axis. IFIT-1 delta Ct values are indicated on the y-axis. Spearman correlation coefficient and P value are shown on the graph. The high R^2 value indicates a closer relationship between RIG-1 and IFIT-1 expression.

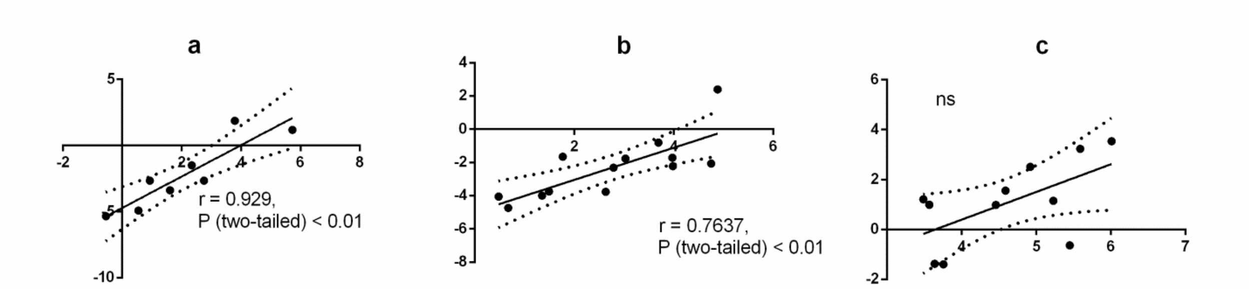

Figure S7. Correlation between IFIT-1 and MDA5 expression with COVID-19 (a), influenza (b), or healthy volunteers (c) at the early phase of illness (3-4 days post symptoms onset). MDA5 delta Ct values are indicated on the x-axis. IFIT-1 delta Ct values are indicated on the y-axis. The elevated R^2 value indicates a closer relationship between RIG-1 and IFIT-1 expression.

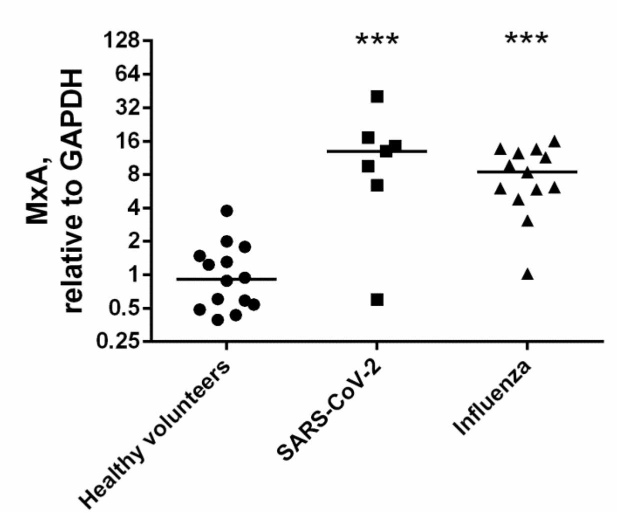

Figure S8 MxA is reliable marker of viral infection expression. Gene expression of MxA in WBCs was analyzed via the ΔΔCt method (relative to GAPDH). Statistical significance (p-value) was determined by Kruskal-Wallis test, followed by Dunn's multiple comparisons test; *** — adjusted P Value < 0.001 compared to healthy volunteers. Data are represented as median.
